# Supplementary material for: Orchard layout and plant traits influence fruit yield more strongly than pollinator behaviour and density in a dioecious crop
Source: PLoS One. 2020 Oct 23;15(10):e0231120. doi: 10.1371/journal.pone.0231120 (PMC7584186; doi:10.1371/journal.pone.0231120)
Supplement: S1 Appendix — However, we note that parameters σm, σf, tm, tf and the proportion of female flower buds in the field exhibit nonmonotic behaviors. Therefore, we conducted additional LHE sampling by truncating the ranges of these parameters to monotonic regions. S1b and S1c Fig depict the monotonicity of the truncated parameter space. The resulting PRCC results for the entire parameter space as well as the truncated parameter spaces are compared in S2 Fig. Parameters for the total number of buds, percentage of female buds, bee density, and handling time are consistently identified as important parameters in all cases. We note that in the truncated case we split the percentage of female flower buds into the cases of 5–76% and 76–96%. In the first half this parameter shows a highly influential positive relationship with predicted yield (large positive PRCC value) and in the second half the parameter is inversely related to the predicted yield. This is as expected as saturating the field with only female buds will eventually cause a decrease in yield. These dynamics are observed in the monotonicity plots as well. (PDF) [file pone.0231120.s001.pdf]

S1 Appendix. Many of the parameters have monotonic relationships with the output measure (S1a Fig) and the PRCC statistics for those are reliable. However, we note that parameters σ_m, σ_f, t_m, t_f and the proportion of female flower buds in the field exhibit nonmonotic behaviors. Therefore, we conducted additional LHE sampling by truncating the ranges of these parameters to monotonic regions. S1b and S1c Fig depict the monotonicity of the truncated parameter space. The resulting PRCC results for the entire parameter space as well as the truncated parameter spaces are compared in S2 Fig. Parameters for the total number of buds, percentage of female buds, bee density, and handling time are consistently identified as important parameters in all cases. We note that in the truncated case we split the percentage of female flower buds into the cases of 5–76% and 76–96%. In the first half this parameter shows a highly influential positive relationship with predicted yield (large positive PRCC value) and in the second half the parameter is inversely related to the predicted yield. This is as expected as saturating the field with only female buds will eventually cause a decrease in yield. These dynamics are observed in the monotonicity plots as well.

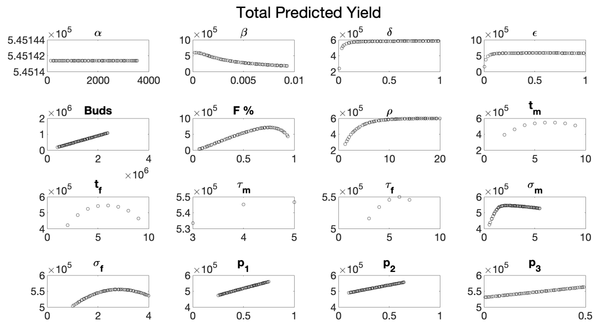

(a)

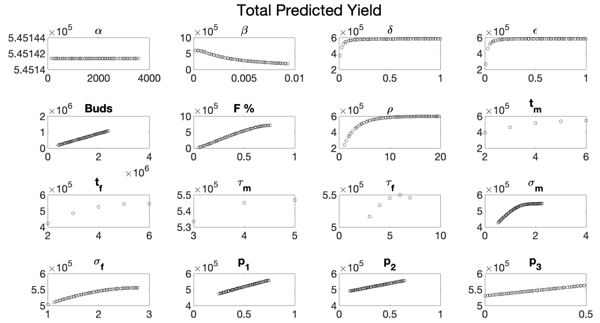

(b)

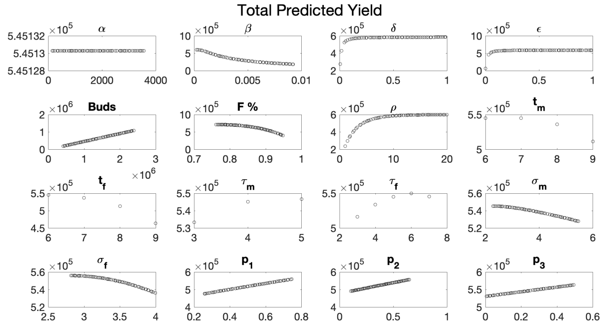

(c)

S1 Fig. Latin hypercube sampling monotonicity plots for the (a) original parameter ranges listed in Table 2 with the percentage of female buds $\in (5\%, 95\%)$, (b) truncated parameter ranges including $\sigma_m \in (.05, 2.27)$, $\sigma_f \in (1, 2.8)$, $t_m \in (2, 6)$, $t_f \in (2, 6)$ and percentage of female buds $\in (5\%, 76\%)$, and (c) truncated parameter ranges including $\sigma_m \in (2.27, 5.5)$, $\sigma_f \in (2.8, 4)$, $t_m \in (6, 9)$, $t_f \in (6, 9)$ and percentage of female buds $\in (76\%, 96\%)$.

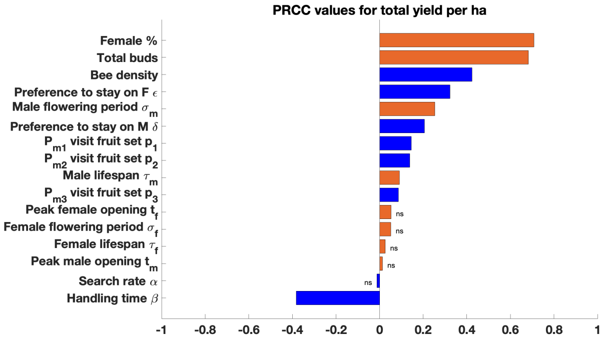

(a)

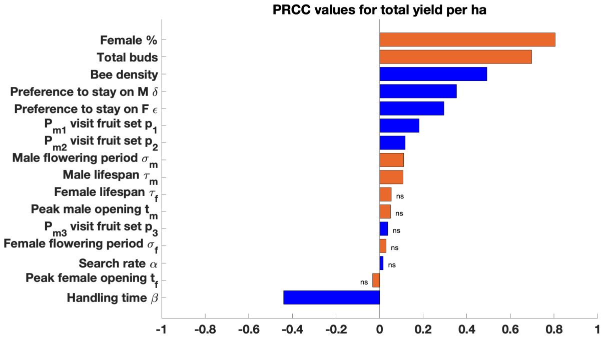

(b)

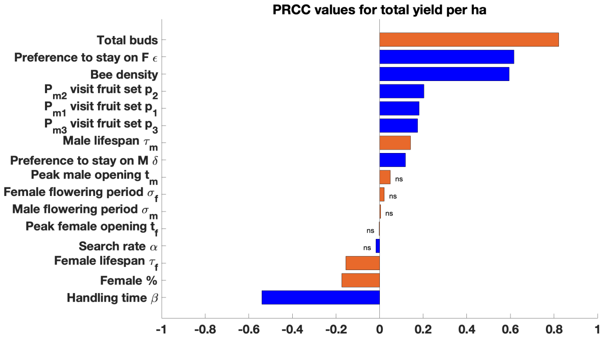

(c)

S2 Fig. Latin hypercube sampling Partial rank correlation coefficient values for each parameter in the (a) original parameter ranges listed in Table 2 with the percentage of female buds $\in (5\%, 95\%)$, (b) truncated parameter ranges including $\sigma_m \in (.05, 2.27)$, $\sigma_f \in (1, 2.8)$, $t_m \in (2, 6)$, $t_f \in (2, 6)$ and percentage of female buds $\in (5\%, 76\%)$, and (c) truncated parameter ranges including $\sigma_m \in (2.27, 5.5)$, $\sigma_f \in (2.8, 4)$, $t_m \in (6, 9)$, $t_f \in (6, 9)$ and percentage of female buds $\in (76\%, 96\%)$. Flower traits are in orange and pollinator traits are in blue.
