# Supplementary material for: Orchard layout and plant traits influence fruit yield more strongly than pollinator behaviour and density in a dioecious crop
Source: PLoS One. 2020 Oct 23;15(10):e0231120. doi: 10.1371/journal.pone.0231120 (PMC7584186; doi:10.1371/journal.pone.0231120)
Supplement: Attachment — Submitted filename: ResponseToReviewers.docx [file pone.0231120.s003.docx]

Adrian G Dyer, Ph.D.
Academic Editor
PLOS ONE

Dear Dr. Adrian G Dyer,

Thank you for your careful review of our manuscript entitled “*Orchard layout and plant traits influence fruit yield more strongly than pollinator behaviour and density in a dioecious crop*” and invitation to submit a revised version addressing the points that arose in the review process. The two reviewers are clearly knowledgeable and provided helpful feedback that has improved the manuscript. Below we list each issue from the reviews followed by with our detailed responses in bold.

**Editor**

I have been able to source two reviews from researchers that have expertise on both modelling and plant pollination systems. Both reviewers see the value in your work, as do I, but also made several suggestions that will significantly improve the clarity of the study for the readership of PLOS ONE. Can you please pay careful attention to all suggestions, and especially work to make the definitions etc much clearer (and more consistent). There were also several points in the manuscript where it was not totally clear where parameter data for models had come from. Please be very careful about defining source(s) all empirical data and how this can be accessed from public domain. This enables other researchers to replicate and extend your research. Data can for example be place within table(s) in manuscript; or use one of the public databases if large data sets are involved. If you had a goal that a good HDR could look up your paper and replicate findings, that is a very good outcome and would help ensure others will use this potentially important finding.

**Response: Thank you for your invitation to submit a revised version. We appreciate the thoughtful suggestions of the two knowledgeable reviewers. Overall, we added extensions of the biological motivations, explanations of typical orchard practices, and several clarifications of model assumptions and parameter values. Below we present detailed responses to each remark.**

**Reviewer #1**

This article presents a model of the honeybee pollination of kiwifruit in order to assess how male/female ratio and honeybee stocking rates impact fruit production in an orchard. The research model is described, results are presented and analysed. The problem is clearly defined, an appropriate model is used, and the potential impact of the results is explained. Overall, this paper addresses an important problem using a relevant and appropriate method.

I think a number of issues still need to be addressed prior to considering whether or not this paper should be accepted for publication. If they can be addressed, then I do think the paper warrants publication and I also believe PLoS ONE is a suitable venue.

**Response: Thank you for your careful review and helpful suggestions to improve our manuscript. We appreciate your attention to details and hope we have fully addressed all your concerns with this revision.**
-----------------------
My main overarching suggestions are:
-----------------------

* that the standard orchard planting ratios be explored and clearly explained to the reader who is unlikely to be familiar with them. These ought to be supported with recent documentation or info. elicited from farmers/growers about current best practice.

**Response:** **As a perennial horticultural crop that can be in the ground for 60+ years and maintain productivity, “recent information” for kiwifruit planting schemes means citations post-1990. Anecdotally, many growers in New Zealand that Plant and Food Research have worked with have the same planting regime they did in the 1980’s, only slightly modified by disease incursion killing mature plants over time. We have included a paragraph in the introduction explaining the growing system and planting ratios, along with a number of citations (see below under “Detailed Remarks”)**

* that the paper be restructured so that the reader finds out, as they read the paper, the info. they need to understand what was done and why it was done using the methods/approaches documented. Currently much info. that is needed to understand the first parts of the paper only appears in the later parts of the paper. Specific details are provided below.
**Response: We restructured to methods section as suggested. We also added a table describing the model variable and parameters to help readers better understand (table 1).**

* that the paper include a careful explanation and justification for the assumption of male/female ratios used in parameterisation
**Response: We added details explaining why we assumed constant ratios when parameterizing the preference parameters.**

* that the paper include a careful explanation and justification of how a bee's flower preference could possibly be independent of flower m/f ratios for an insect foraging in the field.
**Response: We believe that we have clear things up here. While, we assume constant preference parameters, for a bee to remain on a certain type of flower, the probability of moving between flower types incorporates both these preferences, as well as the flower m/f ratios. We carefully described equation 4 and adding in figure 2 to help clarify. We also noticed a couple places throughout the text where we mis-defined the preference parameters (preferences for switching from male to female vs preference to remain on male flowers). We fixed these errors and apologize for the confusion they caused.**

* please label graph axes with units (see below)
**Response: Done.**

* please comment on the utility of the finding about the impact of male opening times to growers. Is it feasible for them to manipulate this trait? Discuss.
**Response: Growers can control male flowering times by their choice of cultivar, as well as pruning and bioregulators (see detailed response below).**

* that the model code be placed online if possible and in accordance with any journal policies if required (e.g. GitHub?)

**Response: We wrote the model in matlab and used matlab’s built in differential equation solvers. We have submitted the code with this revision. We added Supporting Information S2 section to the manuscript: “***The Matlab code for our model was provided as an online supplemental file and is available to download.”*

-----------------------
Some detailed remarks:
-----------------------

Line 7: note, not all animal pollinated plants have *insect* pollinators.
**Response: That is a good point, we changed this word to “pollinators”.**

Line 10: please cite reference for stocking rates 3-8 col./ha.
**Response: The reference for this statement (#4) was in the References section, but not in the text for some reason. This has been corrected.**

Line 17: Explain briefly how this approach could be used to improve conservation practices, e.g. in a sentence or so. Or, drop this sentence altogether.

**Response: We have deleted this sentence.**

Line 26... for the non-kiwifruit-experts among your readership (probably most of us readers!) please provide some detail on orchard layouts, standard planting arrangement, physical characteristics of plants including their extent, leaf cover, number of flowers, pruning regimes etc. Otherwise all this is very hard to get a handle on.
**Response: We agree with the reviewer that the previous draft does jump right in without explaining much about the system. To address this, we have added the following paragraph to the introduction to quickly familiarize the reader with kiwifruit:**

*“Kiwifruit is a deciduous vine, with male and female flowers borne on separate plants (Schmid 1978). Neither sex produces nectar, and the female flowers produce inviable pollen (Schmid 1978), which is high in lipids (Manning 2001), to attract pollinators instead. Plants are typically trained onto a pergola system, with male vines interplanted amongst a larger number of female vines at a 1:3 to 1:8 ratio (Goodwin et al 1999, 2012). Although male cultivars typically have 2-3x more flowers than female cultivars (Testolin 1990), grower planting and pruning regimes ultimately determine the floral sex ratio in orchards. While kiwifruit have a number of pollinating species in their native range (Steven 1988a, b; Yang and Wu 1990), most growing regions rely on honey bees for pollination, representing the vast majority of all flower visitors in the United States (McKay 1978), France (Vaissière et al. 1990), Australia (Howpage et al. 2001), and New Zealand (Clinch 1984; Howlett et al. 2017).”*

Line 34: please, if honeybees are indeed the common managed pollinator of this crop, please provide references to support this (and therefore the choice of honeybees for your model - you only get to this at line 250 currently). Also comment briefly, do bumblebees help? What other pollinators might be playing a role? I see at line 270 you note that the presence of other pollinators may impact honeybee behaviours. For the reader to assess the likelihood of this calling into question the reliability of your results, can you provide your thoughts? Please discuss this in a little detail in the Discussion section also.

**Response: Added paragraph to introduction (see previous comment)**

Line 50: At this point it looks like an essential aspect of your model assumptions is an unlimited supply of pollen on the male flowers. Please comment explicitly.

**Response: This is a limitation of this modelling approach, but to address this our model uses a 4-hour window of pollen-foraging activity to limit the total amount of pollination in a day (this is built in to the visitation rate parameter). Within this window, pollen availability is typically not a limiting factor in kiwifruit due to male flowers having up to twice as many pollen grains than female flowers, and the anthers continued to dehisce over this four hour period. We have added the following sentence at line 76 to explain this:** *“We assume that male pollen availability is not limiting in this scenario; to partially compensate for this short-coming of the model we limit active foraging to four hours per day as captured by the visitation rate parameter, corresponding to field observations (Goodwin 1987).”*

lines 53-63: Please can you make this explanation clearer? E.g. simply saying that a move up from f-3-2-1 occurs by visiting male flowers, and a move down occurs from 1-2-3-f by visiting female flowers, and then referring the reader to the figure would be much simpler.

**Response: We’ve simplified these lines to the following:** *“A pollinator with a high pollen load can move between compartments P_m1_, P_m2_, P_m3_, and P_f_ with subsequence visits to female flowers. Regardless of current pollen loads, whenever a pollinator visits a male flower it completely loads up on pollen and enters the P_m1_ compartment.”*

line 68: Please clarify - honeybees are excellent generalists exploiting a wide range of plant species! Surely they are the exemplar polylectic insect (so not olig.!). Doesn't this mean you need to be more careful about putting the case for your selection of Holling-II? E.g., is the fact they are in an orchard which is mono-culture relevant here since they may have no choice of floral resource? Or is there some other reason your assumption works? Please cite references backing up your claim of appropriateness.

**Response: This is a good point. Geber et al. 2006 suggests that saturating functions of flower densities such as Holling type II functional responses are typical of oligolectic consumers that use only a few plant species and Holling type III responses are typical of generalist consumers that switch between hosts. While honeybees are generalist, here we use a Holling type II response because of the mono-culture orchard environment in the model. We added this clarification to the text.**

EQNs 2a-d. I'm unsure at this point why the equations take the form of the fractions raised to a power. How did you derive this form? (Maybe it is something obvious, sorry if I missed it)

**Response: We provided a clearer explanation of theses functional forms used. In particular we added the following lines;** “*We used these preference parameters to define functional forms representing the probability of a pollinator to visit each type of flower, following the method used in Shaw et al. 2017. These probabilities depend on the ratio of male to female flowers raised to the power of the preference, such that the movement of pollinators between flowers can be written as the following expressions:”* **before the preference equations. Along with these descriptions, we made some figures to help visualize the role the preference parameters and the flower f/m ratio in the orchard. Please see figure 3.**

Line 88: You speak here of flower opening rate. Do you perhaps mean that these times tm and tf are times at which the peak *numbers* of flowers are open? Or is this truly the time at which the *rate* of flowers undergoing the change from open to closed is occurring? (I'm just unsure of your intended meaning in this sentence)

**Response: Here, we do mean the time at which the *rate* of which flowers open is at its peak. To clarify, we reworded this to:** *“… where t_m_ and t_f_ are the times when the opening rates are highest.***”**

Fig 2. refers to a 5x5m orchard. This was initially very confusing to me as I couldn't see why you would model a space so tiny. Only later (cf. line 145) did I realise this fig. caption is the only place 5x5m is mentioned. Can you not instead simply plot this data for the orchard size (1 ha.?) that you actually used for your models? This will save confusing your readers.

**Response: Thank you for the suggestion. We recreated these figures for 1 ha. as suggested.**

Line 104: only below on line 111 does it become clear to me what you mean by this sentence. Please rephrase it to be clear that there is *not* a separate probability parameter or distribution for "fruit set" that is somehow independent of the number of visits. The way 104 is currently worded it seems like this is what you have implemented.

**Response: To avoid confusion, we removed the line 104 and added details to the beginning of the Pollination measurement subsection (previously, line 111).**

Table 1: Can you comment on the fact that the data you have collected in this table spans a variety of scenarios, even decades and (of course) studies? What does this mean for your study that you have drawn across such a wide variety of conditions to extract parameters for modelling a single orchard?

**Response:** **There is no single study that has measured all these parameters simultaneously, which is why we believe modelling to be a useful approach to shine light on the questions we have about the interaction between pollinator and plant factors in determining pollination rates in these orchards. Additionally, all the studies were conducted on the same variety, and numerous studies did collect comparable data for key parameters which gives us the confidence that these parameter ranges are reliable estimates of the biology of the system. We also provide a sensitivity analysis, which explores sensitivity of model outputs across parameter ranges.**

Lines 128-131: This section is very confusing to me. Surely the chance of a M->F or F->M swap is proportional to the number of flowers of each type in visual or short-term exploratory range, *as well as* the preference of the bee? Treating the preference of the bee as the all important factor and treating the problem of availability as independent of this seems to me to be counter to the likely behaviours of foragers in the field (If the bees were in a y-maze and asked to make a decision about M/F I would expect different results.) This issue re-appears at line 184 and I note you are concerned about its importance at line 270. It really seems to me that this is a key parameter needing careful thought and study.

**Response: Thanks for pointing this out. It was is need of clarifications. We added the following text:** *“Experimental observations on pollinator behaviors in environments with equal density of male and female flowers (1:1 ratio, which is typical across planting regimes even when the ratio of male to female plants differs, due to flower pruning practices) reveal that pollinators on male flowers have a 0.957 probability of remaining on male flowers and those on female flowers have a 0.951 probability remaining on female flowers (Goodwin et al. 1993, Howpage et al. 1999 , Broussard et al. 2020). This information was used to help parameterize the preference parameters. Following the experimental conditions, we assume an equal density of male and female flowers and take delta=ln(0.957)/ln(0.5) and epsilon=ln(0.951)/ln(0.5). It is important to note, that while these preference parameters are constant, the probability of switching from flower types depends on these preferences, as well as the open number of male and female flowers, see Equations 4 and Figure 3.”*

[[ Aside: Since neither flower offers nectar, may I ask, what is known about why the bees "want" to visit the female flowers at all? If they *could* tell male from female flower 9I see your note on line 267 that this is possible), may I ask, would they not choose male flowers every time so as to forage pollen? (I'm not familiar with the specifics of bee foraging on this particular crop, I guess many readers won't be either, so please state/reiterate in your paper what you (and the literature) know about why these switching probabilities are as they are.) ]]

**Response: Female kiwifruit flowers produce a pseudopollen that is high in lipids, which are a sought-after resource by honey bees. We have included this information in the new paragraph in the introduction which explains the study system.**

Line 130: You derive some values for preferences based on an equal number of male/female flowers. As a reader I don't know if this is a sensible assumption. My quick and naive googling online reveals: "The proportion of non-fruit bearing, and thus “unreproductive” male plants in the orchard is generally limited, e.g. often 1 male plant to 5-8 females is advised. This limits availability of pollen." (here: <https://www.biobestgroup.com/en/news/the-latest-in-kiwi-pollination>). So now I am left wondering about your derivation. Can you explain this so that the reader can follow why you assumed equal numbers of flowers to compute your parameters? You might also wish to explain why then it is okay for you to vary the M:F ratio in your experiments, without recomputing these parameters (see note above on lines 128-131)? [I note your comment on line 281 that 50:50 ratio is normal... is this based on current practice? The paper you cite is 2012. Is there a newer reference? (Or, if not, is there evidence that nothing has changed from talking to a commercial farmer might help support your claim?)]

**Response: For this study we measured flower number on male and female plants. Male plants have a much higher density of flowers than female plants, so at a plant ratio of 1:8 (M:F), the actual ratio of flowers is approximately 50:50. However, selective pruning can alter these ratios and this is a tool that can be used by growers to manage pollination in their orchards. We have not published this data, but are open to either including this small dataset in this paper or to placing the dataset on BioRxiv if you consider this to be important for this paper. Planting practices have not changed in the last decade, so the 2012 reference does adequately represent current practice. To address this, we have added the following text at line 146 in parentheses:** *“(1:1 ratio, which is typical across planting regimes even when the ratio of male to female plants differs, due to flower pruning practices)”***. We have also added a comment in the introduction mentioning that grower planting and pruning practices will ultimately decide the flower ratio.**

Line 140: Please can you confirm, and cite ref's supporting the idea, that the male and female flowers are both open for the same length of time? Is it true that the pollen from a particular male flower is viable for the same length of time that the stigma of the female flowers are receptive? (This isn't necessarily the case). If not, does your model need to take this into account somehow?

**Response:** **Flower longevity for female and male flowers are separate parameters, and in this model they have different default values (τ_m_ and τ_f_). Different cultivars of male and female flowers do vary in longevity in the field, so growers can select male cultivars to suit the female cultivar they have planted. Our model specifically takes this into account.**

Line 155: You write, "Intuitively... " but actually, haven't you hard-coded 66%, 55% and 22% as the chance that a visit of type 1,2 or 3 will fully pollinate a flower? So this isn't "intuitive", your code is explicitly written to operate this way. Is that correct? Please can you clarify and rephrase if necessary?

**Response: True. We changed the word *Intuitively* to *As expected*.**

I am not an expert on the Parameter Sensitivity analysis approach so I am not able to comment on how well this has been conducted.

Lines 201-202. It wasn't clear from the text above that this variation was going to be explored. Please can you state above that this was something you would investigate? (It seems like a good idea). Can you elaborate, is there actually any means by which a grower might manipulate flowering time of kiwifruit? I.e. could a grower capitalise on this? How? Or is it beyond their control?

**Response: Firstly, we added a paragraph stating the parameters we investigated starting in line 202:**

*“We varied key model input parameters and investigated model predictions with numerical simulations and sensitivity analysis. Model parameters are presented in Table 2. A major model output measure is the predicted yield, which is defined as the number of female flowers per ha that became fully pollinated fruit. A second important model output is the percentage of female flowers that became fully pollinated fruit, defined here as the fruit set. We used numerical simulations to explore variations in flowering dynamics including the percentage of buds that are female and shifts in the duration of time when male and female flowers are both opened (by varying the peak date in male flower opening rate). We also explored variations in pollinators dynamics including bee densities, preference parameters and pollinator handling time****.”***

**Secondly, growers can and do manipulate flowering time. We added a few sentences and citation for this in line 248: “***Pruning and the use of chemical bioregulators are typically used to control the onset and duration of flowering by growers (costa 2010). Over a longer time frame, cultivar selection can be used to ensure adequate overlap of male and female flowering across a range of environmental conditions.”*

Fig 3d) Please match vertical axis scales in all three parts of the figure.
3f) caption... spelling of pollinated.
Fig 4. Please use a numerical value scale that matches the unit (i.e. % not decimals)**.**
Fig. 6 - vertical axis unit = fruit per ha.? Please write it on the graph.
Fig. 7. Caption: Blue dashed line [singular] in (a) depict*s*... and and red dashed lines [which line*s? there's only one line [singular] depict*s*...
Fig. 7(a) Graph: I struggle to read this. Firstly, please place a unit (fruit count?) on the axis. Secondly, aren't the yields per ha. going to be for a specific *pair* of M->F AND F->M values? How do I read a yield for a specific pair of these values off the graph? I can only see how yield corresponds to a single value of EITHER M->F OR F->M switching preference. Or have I really misunderstood what is being shown in this figure? If so, please clarify for me.
Fig. 9 caption (P???) - scrambled text on my PDF copy

**Response: We made these minor modifications. We understand the confusion on Fig.7(a), which is Fig. 8(a) in the updated manuscript. We clarified the meaning on the preference parameters, delta and epsilon throughout the text. We modified this figure caption and added the following text to the results section:** “*It is important to note that Fig.8a explores variations in preference parameters for a constant male to female flower ratio. The actual probabilities of moving between types of flowers is described in Eq. 4 and variations in preference parameters are depicted in Fig. 2 across different orchard conditions*.”

Please check references 16, 24, 38 - they are scrambled or incomplete.

**Response: Corrected, thank you.**

**Reviewer #2**

Recommendation: this article has sufficient potential, with minor revisions.

The authors have used historical empirical data to parameterise their mathematical modelling (delay differential equations with Latin hypercube sampling and sensitivity analysis) to test the relative importance of pollinator behaviour and plant biology, both singly and simultaneously, on fruit yield in kiwi fruits grown in New Zealand. While I understand mathematical modelling is important for simulating the often-complex processes observed in biological systems which can enable effective generation of hypothesis, modelling is often only as good as the data used to create the model. This makes the paper hard to assess the authors claims without critically reviewing the source material used in this model. I would recommend a summary of the empirical data used and extracted in these models. However, I believe the mathematics behind the modelling appears to be sound and nicely incorporates, plant and pollinator data and has the potential to inform kiwifruit growers how to improve yields based on plant ratio/pruning/layout and stocking rate of honeybee pollinators.

The model predicted the following based on such data (from the abstract):
1. At realistic bee densities, the optimal orchard had 65-75% female flowers
2. The most benefit was gained from the first 6-8 bees/1000 flowers, with diminishing returns thereafter
3. Bee density significantly impacted fruit production
4. Plant-based parameters of flower density and male-to-female flower ratio were the most influential

My main concerns about the paper is the decisions and data behind the assumptions and base-line parameters used in the modelling. Generally, I believe the title, abstract and introduction needs to be re-worked so that they are less confusing and flows better. The materials and methods, results and discussion sections are more logical and flow reasonably well. References need to be checked again (some references are incomplete).

**Response: Thank you for your valuable suggestions. Please see our responses below.**

Title
The title makes no mention of the fact that results were obtained by mathematical modelling.
The title does not inform the reader of what type of crop was used (Actinidia deliciosa ‘Hayward’ or A. chinensis?)
The title does not inform the reader what type of pollinator was used (Apis mellifera? Bombus sp.?)
**Response:** **We picked our title with the aim of drawing in a broad readership for our paper. Thus we chose a title that conveyed our central result rather than the details of the approach or study system. We hope this will be appealing to generalists (who might disregard a methods-specific or system-specific paper) and also to specialists (who can find the details of our approach and system in the paper). *Actinidia deliciosa* is an obsolete name, and all cultivars of this larger kiwifruit species are now considered to fall under *Actinidia chinensis*. We have now added the specific names for both the crop and honey bee in the abstract: this was an oversight. If this remains an issue, we could add to the end of the title: “ : a model of honey bees (*Apis mellifera*) pollinating kiwifruit (*Actinidia chinensis*).”**

Abstract
Throughout the paper the authors have used interchangeable wording for the variables and parameters used in this model, making it quite confusing. Ie. Pollinator behaviour / insect behaviour / pollinator abundance / stocking rates / pollen loads / flower handling / flower preference / switch preference and ratio of male to female flowers / densities of male and female flower, percentage of female flowers, plant varieties, plant traits, plant biology, flower phenology, yield, orchard layout etc. In order for this to be less confusing, it may be beneficial to list the exact variables and parameters used, their definition and why you used them. I believe more outcomes from the results section could be incorporated and aspects of the last paragraph of the discussion should be used in the abstract as it is written nicely and provides valuable information for the reader.
**Response: We added clarifications throughout the manuscript, as well as a new table listing and describing the state variable and parameters of the model. The exact parameter values used, are listed in Table 2, as well as in figure captions when needed. We added the following lines to the abstract:** “*The predictive model provides strategies for improving crop management, such as choosing cultivars which have their peak bloom on the same day, increasing the number of flowers with approximately ~70% female flowers in the orchard, and placing enough hives to maintain more than 6 bees per 1000 flowers to optimize yield*.”

Introduction
I think the paper would benefit from additional background information about kiwifruit orchards, varieties, pollinators used, yields etc. What is the current industry standard in New Zealand? Please state clearly and uniformly throughout the text which variables and parameters are being incorporated into the model. Hypothesis / predictions could be expanded and written more clearly.
**Response: This comment was brought up by both reviewers, and we agree that the study system was not explained sufficiently. To clarify, we have added the following paragraph to the introduction:**

*“Kiwifruit is a deciduous vine, with male and female flowers borne on separate plants (Schmid 1978). Neither sex produces nectar, and the female flowers produce inviable pollen(Schmid 1978), which is high in lipids (Manning 2001), to attract pollinators instead. Plants are typically trained onto a pergola system, with male vines interplanted amongst a larger number of female vines at a 1:3 to 1:8 ratio (Goodwin et al 1999, 2012). Although male cultivars typically have 2-3x more flowers than female cultivars (Testolin 1990), grower planting and pruning regimes ultimately determine the floral sex ratio in orchards. While kiwifruit have a number of pollinating species in their native range (Steven 1988a, b; Yang and Wu 1990), most growing regions rely on honey bees for pollination, representing the vast majority of all flower visitors in the United States (McKay 1978), France (Vaissière et al. 1990), Australia (Howpage et al. 2001), and New Zealand (Clinch 1984; Howlett et al. 2017).”*

Materials and methods:
I think it would be beneficial to list all assumptions and why you made assumptions.
Figure 1 description – I understand the explanation is in the main text of the materials and methods section, and this may work for some, however it is not obvious from the description what is going on. At least outline the definitions of Pm1, Pm2, Pm3, Pm4.

**Response: Thank you for these suggestions. We modified the method sections to clarify the assumptions and added in a Table that lists and described state variables and parameters of the model.**

Figure 2. ‘Example simulations of open flowers over time following equations (5) starting with 1500 of each male and female flower buds Bm = Bf = 1500 (modeling 5 meters By 5 meters orchard field) for (a) m = 1, f = 1, tm = 7, tf = 8, m = 5, f = 6 and
(b) m = 2, f = 4, tm = 7, tf = 8, m = 5, f = 6 and
(c) m = 3, f = 3, tm = 4, tf = 5, m = 4, f = 5’
• What is the assumed biological significance of each example?
What unit for yields?
Figures 5 – 5a and 5b – location of the a) and b) descriptions are confusing.
Figure 7 – 7a and 7b – as above
**Response: We added units to the yields throughout all the figures. The example parameter ranges used in figure 2 were arbitrarily chosen just to help visualize these curves. We added the following line to the methods:** *“Here, we chose and varied some parameters values to highlight the role they have in shaping the curve describing flowering dynamics****”***

Results and Discussion
Are written in a clear and logical progression with good discussion.

**Response: Thank you.**
