# Supplementary material for: Orchard layout and plant traits influence fruit yield more strongly than pollinator behaviour and density in a dioecious crop
Source: PLoS One. 2020 Oct 23;15(10):e0231120. doi: 10.1371/journal.pone.0231120 (PMC7584186; doi:10.1371/journal.pone.0231120)
Supplement: Attachment — Submitted filename: ResponseToReviewers2ndRound.docx [file pone.0231120.s004.docx]

Adrian G Dyer, Ph.D.
Academic Editor
PLOS ONE

Dear Dr. Adrian G Dyer,

Thank you for your careful review of our manuscript entitled “*Orchard layout and plant traits influence fruit yield more strongly than pollinator behaviour and density in a dioecious crop*” and invitation to submit a second revised version addressing the points that arose in the review process. .Below we list each issue from the reviewer followed by with our detailed responses in bold.

**Reviewer #1**

This article presents a model of the honeybee pollination of kiwifruit in order to assess how male/female ratio and honeybee stocking rates impact fruit production in an orchard. The research model is described, results are presented and analysed. The problem is clearly defined, an appropriate model is used, and the potential impact of the results is explained. Overall, this paper addresses an important problem using a relevant and appropriate method.

The paper is much improved after its initial revision. I think the paper warrants publication in PLOS1 with only a few minor revisions to tidy things up. These are detailed below.

**Response: Thank you for your careful review. Your helpful suggestions certainly improved our manuscript. We appreciate your attention to details and hope we have fully addressed all your concerns with this second revision.**

-----------------------
My main request is:
-----------------------

Please add a section (or sub-section) on how the model's behaviour was tested... not sensitivity analysis, but how did you do basic checking to ensure it was all functioning as you would expect? E.g. what expected and real-world patterns were you looking to assess against? How did you validate the model's relationship to reality? How did you validate the model's behaviour with respect to your model design?

**Response: We modified the Modeling subsection in the Materials and Method section to include the sub-section header, “Base simulations testing model behaviors”, see page. 9. Here we added some citations to compare the model predictions under baseline parameter values with observations from the literature.**

-----------------------
Some detailed remarks:
-----------------------

ABSTRACT text: "honeybee density per flower" ... "density" seems (to my mind) to conflate two things... i.e. bees / unit-area and bees/flower. Maybe just call it bees/flower as that is how you built the model.

**Response: We modified this to “the number of honey bees per flower”**

Typos below... (please find these using a word search, there are two copies of the paper in the PDF and if I cite line numbers I am bound to be confusing)

>> in *an* orchard
>> femaler !?
>> Here, chose and varied some parameter*'*s values
**Response: Done.**

>> the text, "to partially compensate for this short-coming of the model we limit active foraging to ..." isn't a clear explanation about the pollen issue. BUT the explanation you gave in the document to the reviewers directly in your response document *is* clear... please can you explain the statement in the paper as you explained it in the response to reviewers' document?

**Response: Thank you for this suggestion. We included the following explanation in the text:**

*“We assume that male pollen availability is not limiting in this scenario; to partially compensate for this short-coming of the model we limit active foraging to four hours per day as captured by the visitation rate, corresponding to field observations (Goodwin 1987). This four-hour window of pollen-foraging activity limits the total amount of pollination in a day (built into the visitation rate parameter). Within this window, pollen availability is typically not a limiting factor in kiwifruit due to male flowers having up to twice as many pollen grains than female flowers, and the anthers continued to dehisce over this four-hour period.”*

>> under equation (3): "which has the units of *flowers* per *unit of* time" ...perhaps?

**Response: Thank you, we checked this and the units of equation 3, the visitation rate, is just per time.**

>> under equation (4d): "are on verses the proportion *of* female flowers in the..."
**Response: Done, thank you.**

>> text above equation (6): "type one visit, the visit that results in transitioning a pollinator from group Pm2 to Pm3 139 as a type two visit, and the visit that results in transitioning a pollinator from group Pm3 to Pf as a type three visit".... you can label these on Fig. 2 and refer the reader to the figure for clarification. You might also consider explaining these "types" with Fig. 2, and referring the reader back to it (instead of explaining the types in this section).

**Response: We added labels to figure 2 and simplified this text to, “***We define the visits of pollinators to female flowers that result in pollen deposition as either type one, two, or three, as depicted in figure 2.”*

>> the finding of/around Fig 6 is a little obvious... isn't it? If not, please say why this isn't just trivial (i.e. less male flowers open -> less pollen available -> less fruit set).
**Response: Figure 6 highlights that predicted yield is maximized (with associated fruit set rates above 91\%) when both flower types open concurrently with the same peak opening day. While, it may seem trivial that fruit set is highest when maximizing the number of concurrently opened male and female flowers, it is useful to see how sensitive the yield is to variations in this overlap. We added the following to line 254: “***While shifting the peaks a day apart does not have a huge influence on the predicted yield, a shift of two or three days has significant consequences.”*

Please check references 14, 15, 17 - they are incomplete / truncated.

**Response: Fixed, thank you.**
